# Unintentional effects of environmentally-friendly farming practices: Arising conflicts between zero-tillage and a crop pest, the common vole (*Microtus arvalis*)

**DOI:** 10.1016/j.agee.2018.11.013

**Published:** 2019-02-15

**Authors:** Deon Roos, Constantino Caminero Saldaña, Beatriz Arroyo, François Mougeot, Juan José Luque-Larena, Xavier Lambin

**Affiliations:** a School of Biological Sciences, University of Aberdeen, Tillydrone Avenue, Aberdeen, AB24 2TZ, UK; b Área de Plagas, Instituto Tecnológico Agrario de Castilla y León (ITACyL), Ctra. Burgos km 119, 47071, Valladolid, Spain; c Instituto de Investigación en Recursos Cinegéticos, IREC (CSIC-UCLM-JCCM), Ronda de Toledo s/n, 13071, Ciudad Real, Spain; d Dpto. Ciencias Agroforestales, ETSIIAA, Universidad de Valladolid, Avda. de Madrid 44, 34004, Palencia, Spain; e Instituto Universitario de Investigación en Gestión Forestal Sostenible, Palencia, Spain

**Keywords:** Pest, Outbreak management, Tillage, Land-use

## Abstract

Common voles are a main European facultative, fossorial, farmland rodent pest that can greatly reduce crop yields during population outbreaks. Crop protection against common voles is a complex task that requires the consideration of a set of preventive and control measures within an integrated pest management strategy. A possible option could be to modify farming practices to reduce the availability of refuges for rodents and the damage to crops that they subsequently cause. Farming, however, must simultaneously meet multiple goals including the reduction of the carbon (C) emissions, soil erosion and water use, and the improvement of soil quality. Crop establishment through conservation agriculture strategies, like zero-tillage, would reduce crop management investment, but is also promoted in many regions to reduce C emissions and increase soil organic matter. It could, however, create favourable refuge habitats for fossorial rodent crop pests, like common voles, benefitting from reduced soil disturbance between crop rotations and thus increasing burrow persistence. Assessing the impact that tillage practices, their interaction with different crops and the influence of proximity to potential common vole sources, have on common vole occupancy could provide a valuable tool within an integrated management strategy. Using a 2-ha experimental field with 62 plots 180 m^2^ (each roughly matching common vole home range size) located experimental plots in north-western Spain, we tested how tillage practices, crop type (wheat, barley, vetch, Narbonne vetch, pea and fallow) and distances from possible colonization sources affect field use by common vole during low population density conditions. Our results show that tillage practices have more influence on common vole occurrence (zero tillage > reduced and conventional tillage) than other aspects such as crop type thus supporting the hypothesis that tillage practices play a key role in common vole habitat use.

## Highlights

• Common voles only detected in zero-tilled plots within an experimental design.
• No voles detected in either reduced or conventional tilled plots.
• Zero-tillage offers low disturbance habitat, often with established cover.
• Fallow and vetch plots had highest occupancy, while no voles detected in pea plots.
• Crop nitrogen content not a predictive quality for common vole habitat choice.

## 1 Introduction

The increasing global human population necessitates a commensurate increase in crop yields, all the while doing so in increasingly difficult scenarios presented by climate change (Lobell et al., 2008). However, the role of agriculture in the developed world is more nuanced than simply increasing crop yield (Godfray et al., 2010). Concerns regarding impacts on biodiversity (Tilman et al., 2011), carbon (C) and nitrogen (N) emissions (Smith et al., 2005; Tilman and Clark, 2014), or water retention (Bescansa et al., 2006; Freibauer et al., 2004) must be balanced with the need for increased yields, with no generic win-win scenarios being apparent. In order to achieve these aims, various trade-offs must be managed concerning the competing interests.

One approach to achieve these multiple aims is through the use of agricultural conservation practices which are considered to be environmentally sensitive and economically viable (Soane et al., 2012). An increasingly widely used conservation practice is zero-tillage (whereby seeds are directly drilled into the soil with minimal soil disturbance, accompanied with remaining crop stubble, usually treated with herbicide prior to seeding), which is promoted in many regions in order to reduce C emissions (Lal, 2004; but see Powlson et al., 2014), stabilise higher crop yields (Govaerts et al., 2005) and reduce crop management costs (Lal, 2004). However, such approaches have been suggested to correlate with higher abundance of rodent pests (Heroldová et al., 2017; Witmer et al., 2007) possibly explaining why no-tillage often results in lower yields (Pittelkow et al., 2015b, 2015a).

Rodents are a global cause of varying degrees of crop yield losses, inflicting varying degrees of damage, including complete crop losses locally during high-density pest years (Singleton et al., 1999; Stenseth et al., 2003; Jacob et al., 2014). Difficulties concerning the control of rodent crop pests are inflated since many rodent control methods are largely untested (such as ploughing the field margins to reduce crop colonisation risk), are only locally applied (such as bounty systems, i.e. payments for capturing rodents, Singleton et al., 2003), or make use of conventional approaches which are potentially environmentally risky (such as the use of rodenticides, Buckle and Smith, 2015). In addition, control strategies are often implemented reactively, where the decision to undertake control is based on current densities of pests (Foltete et al., 2016; Heroldová et al., 2017), which may reduce the potential effectiveness of management strategies. As such, preventative management is more likely to be sustainable in the long term and may involve the modification of farming practices.

Some farming practices have previously been identified as being able to reduce *in-situ* pest populations (Jacob, 2003), and may provide a basis to inform strategies which may reduce undesirable effects related to rodent crop pests. For instance, in common voles (*Microtus arvalis*), a fossorial species that is the main vertebrate pest of arable crops in Europe (Jacob et al., 2014), ploughing fields post-harvest was found to reduce the populations dramatically (Jacob, 2003). Two potential causes of for this may be inferred. The first being directly related with mortality and vole disturbance caused by machinery, and the second being that, through destruction of the burrows and removal of remnant vegetation, ploughed fields are no longer attractive habitats or are more difficult to persist in (see Inchausti et al., 2009). Exemplifying the potential of a low disturbance effect, field margins and alfalfa fields are known to be refuges for common voles (Rodríguez-Pastor et al., 2016), both of which are usually left unploughed and undisturbed for protracted periods of time providing cover and habitat stability.

If the second assumption is true, then tillage practice impacts on soil may be important for common vole persistence and/or habitat preference (Bonnet et al., 2013). Practices such as zero-tillage (ZT) may thus presumably increase the likelihood of common vole occupation. The relative soil stability and added residual vegetation cover of ZT compared with other forms of plot management, like reduced tillage (RT) or conventional tillage (CT), involving the movement of soil at varying depths, may further enhance the impact of tillage practices on vole occupancy risk.

Recently, research carried out in the Czech Republic has identified such a trend, where common vole densities were found to be higher in ZT fields, compared with tilled fields (Heroldová et al., 2017). Similarly, common voles in France were found to have disturbed life cycles in fields which experienced disruptive farming activities (such as ploughing) (Inchausti et al., 2009). Testing whether the results observed in temperate regions of central Europe are consistent in other more arid regions with dry, compacted soil is important to inform control strategies of the pest species which occupy large geographical areas. With a species such as the common vole, present from Spain to Mongolia (Yigit et al., 2016) across a correspondingly wide variety of soil types, achieving a broader understanding of common vole occupancy is of particular importance.

The influence of crop type on vole occupancy is similarly important, as well as how these may interact with tillage practices. While studies have investigated the importance of crop type for common vole abundances to various extents, these have either used single crop type characteristics (e.g. Fischer et al., 2017 investigated wheat height) or a small number of crops (e.g. Heroldová et al., 2017 used an interaction between wheat and rapeseed with ZT and tillage, while Rodríguez-Pastor et al. (2016) used alfalfa, fallow and a functional grouping of cereal). Gathering an understanding of how crop types interact with three commonly used tillage practices (ZT, RT and CT) would be of particular interest in an applied setting.

A further consideration, equally important within an applied context, would be the distance between plots and linear habitat features (such as field margins), which usually harbour higher densities of voles, or from other already occupied plots, which could act as sources of colonising voles. Although the issue of distance from source habitat to field are a concern for farmers and managers of official control campaigns, it is not often considered within the applied rodent crop pest literature (but see Rodríguez-Pastor et al., 2016). Understanding the relationship that distance from source populations may have on microscale crop colonisation events is, again, of particular importance as this would inform strategies targeting those features. Currently, control strategies focus on trying to control common voles within linear reservoirs (e.g. field margins, Caminero Saldaña et al., 2015a), or through the use of ploughed strips around field perimeters (acting as a “fire-break” (inferred from Jacob, 2003)), though the effectiveness of both approaches are still insufficiently understood.

We hypothesised that ZT plots would have the highest likelihood of occupation in relation to other tillage practices, and when incorporating crop types, we expected higher occupancy rates in nitrogen-rich crops (Lantová and Lanta, 2009) and fallow, in line with the results of Rodríguez-Pastor et al. (2016). With regards to the likelihood of temporal variation in occupation, we expected occupancy to decrease with distance from source populations, including the field margin harbouring voles as well as nearby occupied experimental plots and/or an influence of differences between crop height. Crucially, we combine these aspects, previously studied in varying degrees of isolation, into an integrated experiment to inform common vole management.

## 2 Materials and methods

### 2.1 Study species

The common vole is a small rodent, weighing approximately 25–30 g (Jacob et al., 2014). As with most microtines, common voles create burrows for nesting, foraging, and predator evasion, with burrows growing in complexity the longer they are occupied (Brügger et al., 2010). Within agroecosystems, common voles have a preference for field margins, alfalfa and fallow fields, moving into cereal crops once populations reach peak densities (Rodríguez-Pastor et al., 2016).

The semi-arid plateau in the central region of Castilla y León (CyL), located in NW Spain is dominated by farmlands: CyL has 3.6 million hectares of arable land, 84% of which is considered to be under rainfed conditions. Predominantly the crops farmed in the region are wheat and barley which are collectively farmed on 2 million hectares. The area was recently colonised by common voles. The colonisation began in the early 1980s with CyL becoming fully colonised by the mid-1990´s (Luque-Larena et al., 2013). The range expansion was associated with an increase in irrigated herbaceous crops, in particular alfalfa (Jareño et al., 2015). By 2014 eight outbreak events had occurred at the regional scale (Luque-Larena et al., 2015, 2013; Rodríguez-Pastor et al., 2017) with claims of substantial damage to crops, mainly in winter cereals, grain legumes and alfalfa.

### 2.2 Study site, experimental design and vole presence estimation

According to wide scale vole abundance monitoring across CyL, vole populations were observed growing rapidly in 2016, culminating in very high densities in field margins in early 2017. However, this high density within field margins never developed into a full outbreak as the voles failed to disperse into fields, likely due to a large scale drought that affected the Iberian Peninsula at that time (see Table 1) (Roos, unpublished). Additionally, in the area where the study took place, voles, including those in field margins, never showed a marked increase and the population was considered to be at relatively low abundance (Roos, unpublished.).

**Table 1:** Meteorological data collected from the Finca Zamadueñas weather station. Retrieved from InfoReigo.org.

Period	Mean daily temperature	Mean daily humidity	Mean daily precipitation
May 1^st^ to June 30^th^, 2010-2016	14.8 °C	64.2 %	1.1 mm
May 1^st^ to June 30^th^, 2017	17.2 °C	55.7%	0.6 mm

**Fig. 1:**
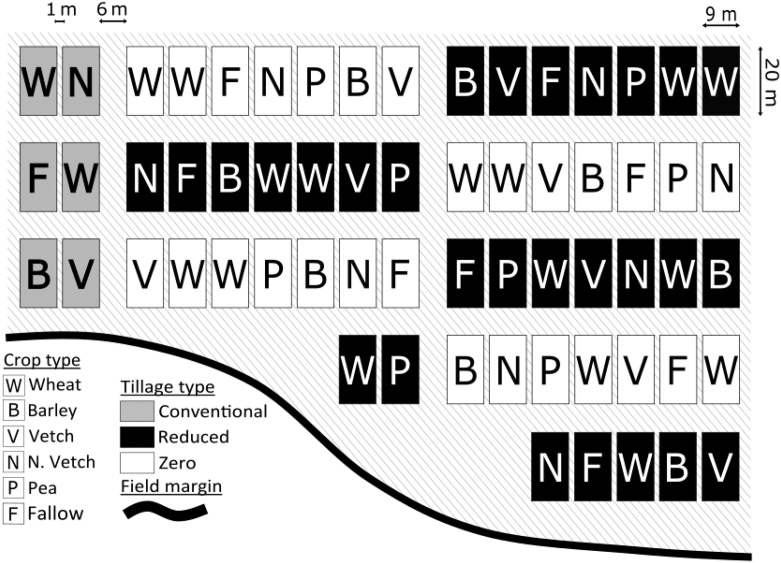
Map of experimental plots. The solid black line indicates the field margin. Tillage types are represented by grey shading (CT), black shading (RT) and white shading (ZT) background within a given plot. Crop types are represented by “W” (wheat), “B” (barley), “V” (vetch), “N” (Narbonne vetch), “P” (pea) or “F” (fallow). Grey diagonal lines represent the ploughed areas between plots.

The research was carried out in 2017, in the last week of April, May and June in a 2-ha experimental field in Zamadueñas, Valladolid (41° 39' 8” N, 4° 43' 24” W, elevation 690 m.a.s.l.). The experimental field is part of an ongoing investigation testing different soil management practices and crop rotations in a context of sustainable agriculture. The experimental field was separated from an adjoining field margin by a strip of land measuring between 3 m to 35 m (see Fig. 1). This strip was ploughed in the previous winter with a harrow disc at a depth of 10 cm to keep these areas of land devoid of vegetation. The field margin was the only nearby habitat, other than the experimental field, with plant cover and vole presence during our research in a radius of ∼90 m from the experimental site.

The experimental field contained nine blocks of plots arranged in a systematic layout using three tillage practices (each block has been continuously managed by the same tillage practice since 2004): ZT (n = 4 blocks, 28 plots; one block was divided in two due to the shape of the field), RT (n = 4 blocks, 28 plots) and CT (n = 1 block, 6 plots; in a 2 × 3 plot arrangement). Within the experimental field, RT block plots were ploughed before seeding with a chisel at a depth of 10 cm, whereas ploughing in the CT block plots, was carried out to a depth of 30 cm using a moldboard plough. No ploughing was applied in ZT plots. Before sowing, ZT and RT blocks were treated with glyphosate 36% at 2 kg/ha. No other pesticides were applied.

Common vole burrows have been described to be 12.6 cm below ground on average, with a maximum depth of 25 cm (Brügger et al., 2010). Thus, RT and CT may penetrate deep enough to disrupt vole burrows, though CT is more likely to destroy entire burrow complexes.

Within each block, seven experimental plots (six in CT block) were randomly allocated one of six crop types; wheat (*Triticum aestivum*), barley (*Hordeum vulgarei)*, vetch (*Vicia sativa*), Narbonne vetch (*Vicia narbonensis*), pea (*Pisum sativum*), and fallow, with wheat replicated twice per block (CT did not have a pea crop treatment). Crops used in the previous year were similarly recorded (see Table 3 in Supplementary Material). Each experimental plot measured 20 m by 9 m. Crop height (cm) was estimated for each plot at each time point (see Table 4 in Supplementary Material).

Within blocks, plots were separated by 1 m, while separation between blocks was 6 m. Both between plots and between blocks, the space was ploughed with a harrow disc at a depth of 10 cm (Fig. 1) after sowing. No vegetation was observed between blocks and plots during data collection.

The size of the plots (180 m^2^) roughly matches the largest measured average home range size of common voles (30 to 202 m^2^) (Briner et al., 2005; Jacob and Hempel, 2003), and as such plots were assumed to be large enough to be (partially) occupied by voles. Common voles are estimated to disperse between 76 m–110 m per generation depending on sex (Gauffre et al., 2009, 2008) though alternative estimates suggest that within hours or a few days, voles can disperse several hundred meters or a few kilometers (Schweizer et al., 2007). As such voles were assumed capable of dispersing to and colonising vacant experimental plots, though this would be most pronounced during periods of high population growth (Andreassen and Ims, 2001).

The experimental plots were surveyed for signs of vole presence three time in the last week of April, May and June 2017. The experimental field was surveyed in consecutive 9 m wide transects, each beginning from the field margin in 3 m paced increments, resulting in the entire 2 ha surface being surveyed. In total, 1478 sections were surveyed over the three sampling periods. 668 of these were in ZT plots, 671 in RT and 139 in CT. Additionally, the field margin was surveyed at the same time as the field using the same transect design. In every 3 m x 9 m section of a transect signs of vole presence were noted. Signs included: burrowing complexes (three or more burrows), fresh latrines, fresh vegetation clippings, fresh digging activity, and runs accompanied by areas of damaged vegetation surrounding a burrow complex of three or more entrances. These indicators are routinely used in the study region (Caminero-Saldaña et al., 2015b,c; Jareño et al., 2014) and are similar to those used elsewhere (e.g. Heroldová et al., 2017). For this study, one or two burrows in a section were not considered as a sign of fresh activity as a burrow may persist for long periods of time despite no voles being present.

### 2.3 Statistical analysis

#### 2.3.1 Spatial variation analysis

For each experimental plot, the proportion of occupied sections in each plot per survey (weighted by the total number of sections) was calculated and used as the response variable in Generalised Linear Mixed Models (GLMMs) analysis. To calculate proportion of usage, for each section within a plot the presence of any fresh signs resulted in that section being considered occupied. Then, the total number of occupied sections per plot was summed for the respective plot and divided by the total number of sections in a plot to give a proportion of usage per plot, weighted according to the number of sections (the latter varied among plots because the pacing out of 3 m was measured by length of stride and was therefore imprecise).

We modelled proportion of vole plot occupancy using binomial distribution GLMMs. The proportion of occupancy (a two vector response variable; number of occupied sections per plot / total number of sections per plot) was considered in relation to: tillage practice and its interaction with crop type; crop type interacting with crop height (crop height was only included as an interaction due to height being crop specific resulting in it being confounded if included as an additive effect, see Table 4 in Supplementary Material); crop type in the previous year (which may have affected persistence in plots) and distance from the field margin. We included experimental plots nested within blocks as a random effect to account for the repeated measures in each plot (reflecting the nested experimental design) as well as unexplained variation between blocks and plots. Plot nested within month was also tested as an alternative random effect structure. Model selection was carried out using single term deletion and subsequent ΔAIC values. GLMM analysis was carried out in R v3.4.1 (R Core Team, 2017) using the *lme4* package (Bates et al., 2015).

#### 2.3.2 Temporal variation analysis

Simple multi-season occupancy models were used to determine initial occupancy (Ψ = the probability that a plot was occupied at the first sampling occasion), colonisation (γ = the probability that voles will colonise a previously unoccupied plot), extinction (ε = the probability that a plot will no longer have voles where previously they present) and detection (p = the probability that if voles were present in a plot, it was correctly identified as occupied) rates per plot and thus the extent of temporal variation during the study period. Each plot was assigned a 1 (occupied) or a 0 (unoccupied) for each survey month, depending on if any sections within a plot had vole activity (e.g. 011 denotes absence in April, and presence in May and June, respectively). The null model, assuming constant initial occupancy Ψ(.), colonisation γ(.), extinction ε(.) and detection p(.) rates, was compared to the full model which used the following: Ψ explained by tillage type, crop type and distance from field margin; γ explained by month and number of occupied adjacent plots (to account for colonization being dependent on distance from already occupied plots); ε as constant; and p determined by crop type. Values for occupancy rates in later time periods and lambda values for transition periods were derived as part of the analysis. Model selection was carried out using single term deletion and subsequent ΔAIC values (Burnham and Anderson, 2004). Occupancy modelling was carried out using the Presence software, version 12.7 (Hines, 2006).

## 3 Results

### 3.1 Spatial variation

#### 3.1.1 Tillage practice

**Fig. 2:**
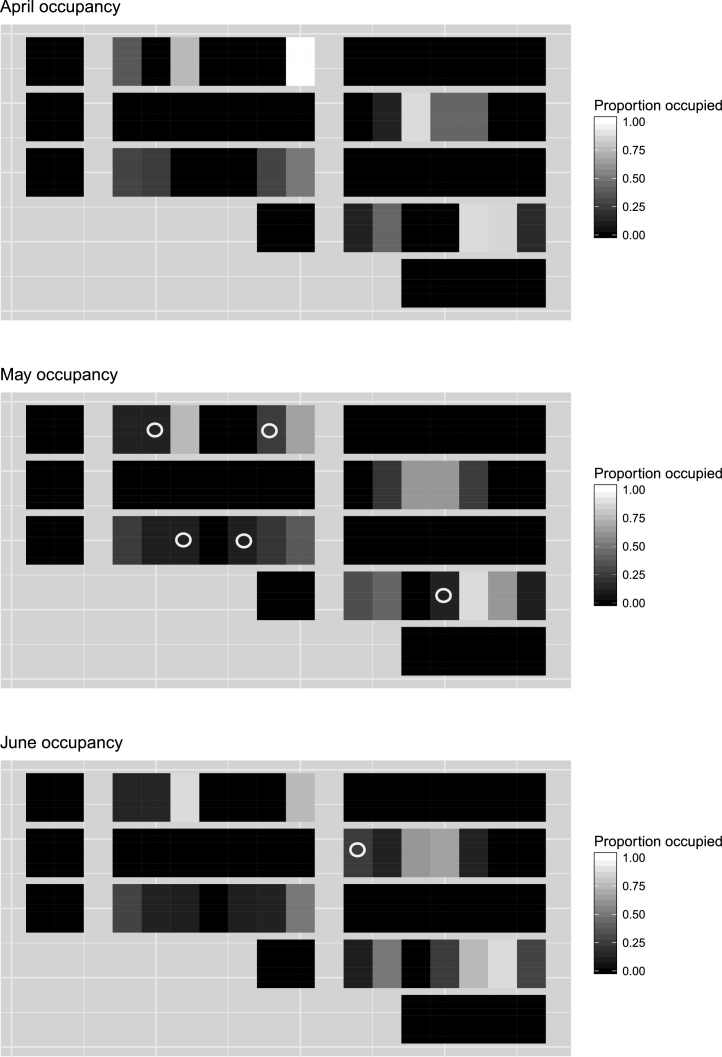
Level plots showing the proportion of vole usage (number of occupied 3 m x 8 m sections / total number of sections per plot) for each month of data collection. Six colonisation events were detected between April to May and June and are highlighted with black circles and white borders.

Common vole activities were only detected in ZT plots (51 of the 84 ZT plots), with no sign of voles detected in the CT block nor the four RT blocks (see Fig. 2). Similarly, for pea plots, regardless of tillage practice, no common voles were detected (see Table 2 for summary).

**Table 2:** Summary of plots with voles detected per month by tillage type and crop type. Note that this does not take detection probability into account. ^*^wheat was replicated twice per block. ^+^no pea treatment was used in CT. (see Fig. 1 for experimental field schematic).

Factor	Number of plots occupied			Total number of plots
	*April*	*May*	*June*	
*Zero Tillage*	17	21	21	28
*Reduced Tillage*	0	0	0	28
*Conventional Tillage*	0	0	0	6
Wheat^*^	5	7	8	18
*Barley*	2	4	3	9
*Vetch*	4	4	4	9
*Narbonne vetch*	2	2	2	9
*Fallow*	4	4	4	9
*Pea ^+^*	0	0	0	8

The complete absence of voles in plots other than ZT and pea plots (Table 2) precluded a global analysis with all the tillage regimes, resulting in GLMM failing to converge due to perfect separation. We therefore further investigated vole presence variation focusing within ZT plots. Accordingly, the GLMM models were fitted excluding data from CT, RT and pea crop plots.

GLMM model selection resulted in the model with only crop type giving the lowest AIC value, while the model with both crop type and distance from the margin had a ΔAIC of +2.0 and so could be considered as equally good (see Table 3 for GLMM coefficient summary). An interaction with crop height and crop type was not retained (ΔAIC +11.52) and the previous years crop type was excluded due to being confounded with the current crop type (crop type rotation was not randomised). We therefore continued with the model with crop type and distance from margin to investigate the influence of the three hypothesised drivers of common vole distribution.

**Table 3:** Coefficient table, in logit, for the generalised linear mixed effects model.

Fixed Effects	Parameter Estimate	Std. Error	Z-value	P-value
*Barley*	−1.412	0.671	−2.10	0.035
*Fallow*	1.791	0.715	2.50	0.012
*Narbonne Vetch*	−0.606	0.761	−0.80	0.425
*Vetch*	2.127	0.715	2.97	0.003
*Wheat*	−0.533	0.639	−0.83	0.404
*Distance from field margin*	−0.001	0.007	−0.06	0.949

#### 3.1.2 Crop type

**Fig. 3:**
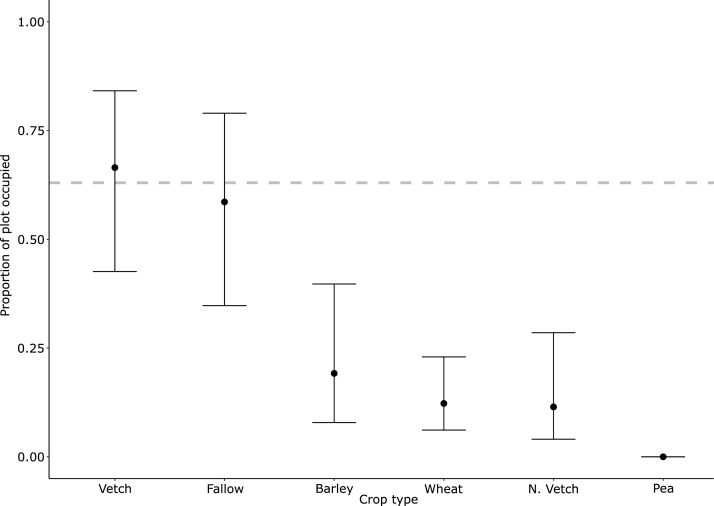
Predicted proportion of plot occupied by common voles according to crop type within ZT experimental plots with 95% CI. The dashed grey line represents the naïve 3-month average proportion occupied in the field margin. N. Vetch shortened from Narbonne vetch.

Spatial variation, as explained by crop type, showed higher proportion of usage for vetch (0.67, 0.43 – 0.84 95% CI, Z = 2.97) and fallow (0.58, 0.35 – 0.79 95% CI, Z = 2.5), with barley (0.19, 0.08 – 0.40 95% CI, Z = -2.06), wheat (0.12, 0.06 – 0.23 95% CI, Z = -0.83), and Narbonne vetch (0.11, 0.04 – 0.29 95% CI, Z = -0.80) having lower proportion of usage (Fig. 3). As stated above, pea plots were never occupied.

#### 3.1.3 Distance from field margin

The proportion of usage in each ZT plot, as explained by distance from the margin, showed no significant trend with increasing distance from margin (-0.0002 ± 0.032, Z = -0.032, P = 0.97).

#### 3.1.4 Random effects

The nested random effect terms, block and plot, explained 0.76 of the variance and block explained 0.03 of the variance, suggesting that while there was variation between plots, the interaction between blocks and plots explained a greater proportion. Alternative random effects, with plots nested in month, were tested but had higher AIC scores and explained less variation, and thus were not included in the final model.

#### 3.1.5 Occupancy variation in field margin

**Fig. 4:**
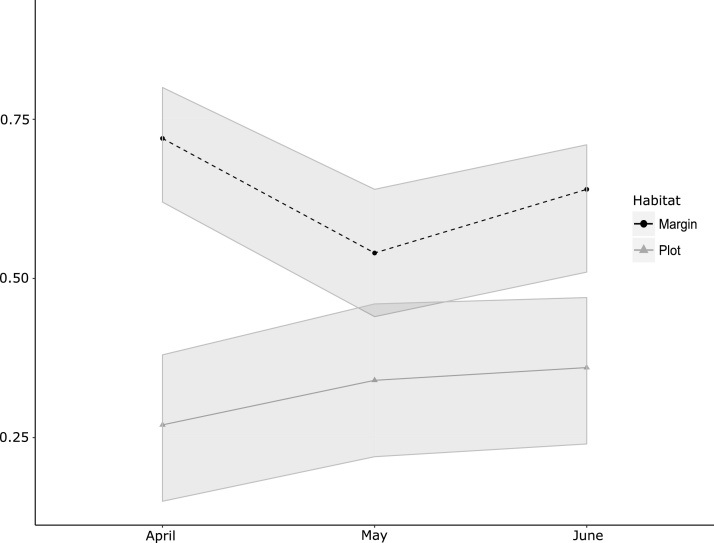
Experimental plot and margin occupancy rate. Plot estimates from multiseason occupancy modelling. Margin occupancy rates are naïve estimates of the proportion of occupied sections in the margin by month. The grey ribbons represent 95% CI around the occupancy rate estimates. Occupancy rates for May and June within plots are derived estimates.

The field margin harboured a common vole population with an average naïve occupancy estimate of 0.63 over the three months (calculated as the number of sections occupied / total number of sections surveyed in the field margin) varying by month from 0.72 (0.62 – 0.80 95% CI) in April to 0.54 (0.44 – 0.64 95% CI) in May and 0.61 (0.51 – 0.71 95% CI) in June (Fig. 4). These values were similar to those observed in vetch and fallow plots within ZT blocks (see above).

### 3.2 Temporal variation

Occupancy model selection resulted in the Ψ(.), γ(Month), ε(.), p(.) model being selected, though the null model (Ψ(.), γ(.), ε(.), p(.)) had a ΔAICs of <2 and so could not be differentiated. In other words, the results suggested that initial occupancy was constant and not explained by tillage type, crop type or distance from field margin, whereas colonisation was only explained by month but not by the number of adjacent occupied plots. Occupancy models were not able to converge when including tillage type, crop type, and number of adjacent occupied plots as variables due to the aforementioned separation. Failure to converge through the inclusion of number of adjacent occupied plots in the occupancy models was likely due to this equating to the number of adjacent ZT plots leading to separation (i.e. an occupied adjacent plot is conditional on it being both occupied, but also the plot being ZT as ZT plots were the only plots occupied).

The results of the final model showed an initial occupancy rate of 0.27 (0.15 – 0.38 95% CI) in April, slightly increasing to 0.34 (0.22 - 0.46 95%CI) in May and 0.36 (0.24 - 0.47 95% CI) in June (Ψ for May and June were derived estimates) (Fig. 4 and see Fig. 2 for per plot vole usage for each month). Additionally, colonisation rates during the study were 0.10 (0.01 - 0.20 95% CI) between April and May, dropping to 0.02 (0 - 0.07 95%CI) between May and June (see Fig. 2 for colonisation events). Derived lambda rates (rate of change in site occupancy) gave values of 1.29 (0.94–1.63 95%CI) between April and May, and 1.04 (0.94–1.15 95% CI) between May and June. Extinction rate per site was consistently estimated at 0 (0 - 0.13 95% CI) for both April to May and May to June transition periods. Detection rate was estimated at 0.97 (0.83–1.00 95% CI).

## 4 Discussion

Within our experimental settings common voles, at low densities, were only found to occupy ZT plots, and never RT nor CT. This may be the result of voles only being present in plots that had not been ploughed prior to the start of our study. Additionally, voles occurred at a higher prevalence in vetch and fallow.

While occupancy analysis showed that the population expanded, from 0.27 occupancy in April to 0.36 in June, and plot colonisation occurred with rates of 0.10 from April to May and 0.02 from May to June, this was limited and the population may be considered to have been broadly static from May, reflecting a likely aborted outbreak event (lambda decreasing from 1.29 to 1.04) as observed regionally. While colonisation rate varied temporally, we found no evidence that colonisation, nor the proportion of plot usage, was affected by distance from field margin, nor were we able to test the influence of number of adjacent occupied plots. Given that we do not have abundance estimates prior to seeding, we were not able to determine the relative importance of colonisation from the field margin or persistence in plots from the previous season to explain occurrence in ZT plots in our first sampling session. However, during the period when sampling occurred, it seems more likely that colonisation occurred from already occupied plots rather than from the field margin (see Fig. 4, Fig. 2).

Caution must be taken with these results owing to the small number of colonisation events which occurred during the study; however, our results match those of Heroldová et al. (2017) (see also Witmer et al., 2007) and may provide circumstantial evidence that the rodent pest species have a preference for ZT fields. Possibly the most likely cause of occupancy in ZT alone, and not RT or CT plots, is the combined persistence from previous year populations and colonisation into unoccupied ZT. In fact, with a species which is controlled, a farming practice which may increase both persistence and preference should warrant further research, as the potential for ZT fields to enable persistence and encourage colonisation has worrying implications for management.

Owing to the small-scale nature of the experiment, caution must be taken if, or when, results from this study are extrapolated to landscape scales. For instance, although we have no data on abundance or damage, common voles were believed to colonise the experimental field in 2007, and practitioners at that time and subsequently in 2014 (during a period of high regional vole abundance) noted that pea crops in the experimental field were disproportionately damaged (Caminero Saldaña, unpublished), though this may reflect pea crops being more susceptible to damage as the meristem is located at the top of the plant (Yaxley et al., 2001). Regardless, similar results regarding tillage have been observed by Heroldová et al. (2017) and crop type by Rodríguez-Pastor et al. (2016), suggesting that the patterns seen in this study are consistent with observations at larger landscape scales. An additional strength of this experiment, however, has been to investigate a wider variety of both tillage practices (all three of the commonly used methods; ZT, RT and CT, where Heroldová et al. (2017) only used ZT and tillage) and crops (Heroldová et al. (2017) considered winter wheat and winter rape, while Rodríguez-Pastor et al. (2016) considered fallow, alfalfa and a functional grouping of wheat and barley). This has allowed us to gather greater insight into the relative importance of a wider array of factors determining the occurrence of common voles. For instance, the higher occurrence of voles in vetch and low occurrence in Narbonne vetch and pea is surprising as this rejects the hypothesis that the voles would occur at higher rates in legume crop types (Lantová and Lanta, 2009), indicating that functional groupings may not be appropriate for common voles with certain crop types.

While we expected that protein-rich legumes would have had the highest occupancy, our results show the opposite, with Narbonne vetch plots having lower occupancy despite having higher crude protein content; Narbonne vetch has on average 234 ± 7 g kg^−1^ forage dry matter crude protein (Hadjipanayiotou, 2000) compared to vetch which has 209 g kg^-1^ (Mikić et al., 2014). A potentially exciting explanation could be that Narbonne vetch is able to repel common voles similar to how another legume is able to repel herbivory (Baldwin et al., 2018). Indeed, previous research has identified a chemical component (γ-Glutamyl-S-ethenylcysteine, GEC) apparently specific to Narbonne vetch, that appears to act as a repellent to monogastric herbivores (Enneking et al., 1998). If the results here relate to this repellent potential of Narbonne vetch, then this may offer tentative evidence that an alternative crop could be used by farmers in the region during periods when an outbreak appears likely. However, caution must be made in utilising this method, as even though Narbonne vetch may be unattractive, during high densities the need to feed may compensate and overwhelm the repellent.

In any case, it is also important to mention that our study design did not allow separating the effect of either previous crop or crop height, as these were confounded with crop type. In both fallow and vetch, the crop types where higher occupancy was detected, had barley as precedent crop (see Table 3 in Supplementary Material). Additionally, all the new colonization events during our study occurred into cereal plots, where vegetation was higher than in other crop types (see Table 4 in Supplementary Material). Rodríguez-Pastor et al. (2016) found evidence that voles were less likely to be present when cereal height was low. Further studies should specifically assess whether previous crop type or vegetation cover are an important additive factors explaining occurrence or colonization.

The immediate implication of our results is that tillage practices must be taken into account when carrying out monitoring and in future work attempting to manage fossorial rodents. Crop type has previously been viewed as the dominant farm specific variable when predicting common vole occupancy (see Foltete et al., 2016), with tillage type broadly not being considered (but see Heroldová et al., 2017; Witmer et al., 2007), though recent studies have begun including additional farming practice variables when studying the pest (Fischer et al., 2017; Jug et al., 2008; Rodríguez-Pastor et al., 2016). More broadly, our results show the importance of relating farming practices in general, rather than focussing on crop type alone, to vole habitat use. While our results agree that crop type is important and must be retained in monitoring efforts, tillage practice has been shown to be a greater predictor of vole presence, necessitating the inclusion of tillage practice into monitoring and, eventually, into efforts aimed at predicting common vole outbreaks.

Other factors, not assessed in our experiment, similarly warrant inclusion in future research such as the use of irrigation, which is important in arid regions for increasing crop height and vegetation cover, which may soften soil allowing easier burrow creation, and allows the expansion of semi-permanent crops such as alfalfa. Alternatively, irrigation could be considered as a control strategy, using it to flood burrows and, consequently, drowning voles or destroying the burrow network.

If it is possible to predict common vole outbreaks then the results presented here, combined with Rodríguez-Pastor et al. (2016) and Heroldová et al. (2017), would suggest that during outbreak-likely periods switching from ZT to a tillage form that has at least some soil movement, such as RT, may help to reduce potential common vole sources and thus the outbreak or crop damage risk. Additionally, although further experimental research is needed, our results suggest that switching crop types (e.g. to Narbonne vetch) could also help. Similarly, the lack of colonisation events in our study into plots separated by 6 m of ploughed ground opens the question of whether the maintenance of strips without vegetation between crops and vole reservoirs (e.g. field margins) could be used as a preventative method to reduce the colonisation of voles into fields. Further studies and analysis of such management approaches may prove valuable for farmers.

## 5 Conclusion

Our results show that generalities may be drawn between arid regions of common voles geographic range and those from temperate regions (Heroldová et al., 2017). However, we emphasise the need to better establish the links between a variety of farming practices holistically, including crop type, tillage type and distance from source habitats, especially when attempting to manage crop pests. Failure to do so risks ignoring important factors determining risks of crop damage and may lead to inefficient management plans. Recent research has moved towards this end with work showing the importance of tillage, both for biodiversity (Barré et al., 2018) and pest distribution (Heroldová et al., 2017), as well as crop type and distance from source populations (Rodríguez-Pastor et al., 2016). How such information is taken into account when attempting to manage pests is not apparent, especially when taking into account the various nuanced aims of farming as well as the trade-off between conservation benefits of ZT (Barré et al., 2018) and pest risk.
