# Supplementary material for: Unintentional effects of environmentally-friendly farming practices: Arising conflicts between zero-tillage and a crop pest, the common vole (Microtus arvalis)
Source: Agric Ecosyst Environ. 2019 Feb 15;272:105–13. doi: 10.1016/j.agee.2018.11.013 (PMC6360522; doi:10.1016/j.agee.2018.11.013)
Supplement: Supplementary file 1 [file mmc1.docx]

**Supplementary Information**

Table 3. Summary table of crop rotations in year proceeding data collection (2016) and during data collection (2017).

| Crop rotation  in 2016 | Crop rotation  in 2017 |
| --- | --- |
| Fallow or Vetch | Wheat |
| Wheat | Barley |
| Barley | Vetch |
| Rye | Narbonne Vetch |
| Barley | Fallow |
| Wheat | Pea |

Table 4. Summary table of mean crop heights averaged over the three months.

| Crop | Mean height (cm) | ±SE |
| --- | --- | --- |
| Barley | 22.8 | 0.4 |
| Wheat | 25.2 | 0.5 |
| Pea | 11.6 | 0.2 |
| Vetch | 8.1 | 0.2 |
| Narbonne vetch | 10.0 | 0.2 |
| Fallow | 4.3 | 0.4 |
